# Area-Level Socioeconomic Inequalities in Adiposity among Older Adults: the Moderating Effect of Neighborhood Walkability

**DOI:** 10.1007/s11524-026-01102-1

**Published:** 2026-06-12

**Authors:** Antoni Colom, Napoleón Pérez-Farinós, Francisco Javier Barón-López, Maurici Ruiz-Pérez, Marc Dominguez Mallafré, Xavier Delclòs-Alió, Antonio Garcia-Rios, Aurora Bueno-Cavanillas, Francisco J. Tinahones, Víctor Urbano-Fernández, José J. Gaforio, Jordi Salas-Salvadó, Montserrat Fitó, Julia Wärnberg

**Affiliations:** 1 Nutritional Epidemiology and Cardiovascular Pathophysiology Group, Health Research Institute of the Balearic Islands (IdISBa), Palma, Spain; 2 Movement, Brain and Health (MOBhE) Research Group and Department of Nursing, University College Alberta Giménez, affiliated to, Comillas Pontifical University, Palma, Spain; 3 Molecular Biology and One Health Research Group (MolONE), University of the Balearic Islands, Palma, Spain; 4 EpiPHAAN Research Group, Department of Public Health, School of Medicine, University of Málaga, Boulevard Louis Pasteur S/N, Málaga, 29071 Spain; 5 Instituto de Investigación Biomédica de Málaga, (IBIMA Plataforma Bionand), Málaga, Spain; 6 Centro de Investigación Biomédica en Red Fisiopatología de la Obesidad y Nutrición (CIBEROBN), Instituto de Salud Carlos III, Madrid, Spain; 7 Geographic Information Systems and Remote-Sensing Service-SSIGT, University of the Balearic Islands, Palma, Spain; 8 Grup de Recerca en Anàlisi Territorial i Estudis Turístics (GRATET), Departament de Geografia, Universitat Rovira i Virgili, Vila-Seca, Spain; 9 Department of Internal Medicine, Maimonides Biomedical Research Institute of Cordoba (IMIBIC), Reina Sofia University Hospital, University of Cordoba, Cordoba, Spain; 10 Department of Preventive Medicine and Public Health, University of Granada, Granada, Spain; 11 Centro de Investigación Biomédica en Red Epidemiología y Salud Pública (CIBERESP), Instituto de Salud Carlos III, Madrid, Spain; 12 Instituto de Investigación Biosanitaria IBS-Granada, Granada, Spain; 13 Department of Endocrinology and Nutrition, Instituto de Investigación Biomédica de Málaga (IBIMA), Hospital Universitario Virgen de La Victoria, Málaga, Spain; 14 Centro de Salud Las Palmeritas. Distrito Sanitario Atención Primaria Sevilla, Seville, 41013 Spain; 15 Department of Medicine, Universidad de Sevilla, Seville, Spain; 16 Departamento de Ciencias de la Salud, Instituto Universitario de Investigación en Olivar y Aceites de Oliva, Universidad de Jaén, Jaén, Spain; 17 Departament de Bioquímica i Biotecnologia, Institut d’Investigació Sanitària Pere Virgili (IISPV), Alimentació, Nutrició, Desenvolupament i Salut Mental ANUT-DSM, Universitat Rovira i Virgili, Reus, Spain; 18 Unit of Cardiovascular Risk and Nutrition, Institut Hospital del Mar de Investigaciones Médicas Municipal d’Investigació Médica (IMIM), 08003 Barcelona, Spain; 19 EpiPHAAN Research Group, Department of Nursing, School of Health Sciences, Universidad de Málaga, Málaga, Spain

**Keywords:** Walkability, Socioeconomic status, Older adults, Obesity, Urban health inequalities, Built environment

## Abstract

Socioeconomic disadvantage is associated with higher obesity risk in older adults, but whether walkable neighborhoods modify these inequalities remains unclear. We examined whether neighborhood walkability moderates the association between area-level socioeconomic status (SES) and adiposity among older adults with overweight or obesity living in Mediterranean cities. This cross-sectional study analyzed baseline data from 1286 urban-dwelling adults aged 55 to 75 years with overweight or obesity and metabolic syndrome residing in six Andalusian cities. Area-level SES was assessed using the 2011 Spanish Deprivation Index, and neighborhood walkability was derived from an open-source index incorporating population density, street intersection density, and access to daily amenities. Adiposity outcomes included waist circumference, waist-to-hip ratio, and a body shape index, all objectively measured. Two-level linear regression models, with individuals nested within census tracts, evaluated SES-walkability interactions while adjusting for demographic and lifestyle factors. No overall differences in adiposity were observed between higher- and lower-SES neighborhoods. However, walkability significantly modified the SES-adiposity association. In highly walkable and dense neighborhoods, participants living in more deprived areas had larger waist circumference and higher body shape index compared with those in less deprived areas, indicating a steeper SES gradient in central adiposity. In low-walkability neighborhoods, SES differences in central adiposity were minimal. No interaction was observed for body mass index. Highly walkable urban environments may therefore amplify socioeconomic inequalities in abdominal obesity among older Mediterranean adults with overweight or obesity. Urban planning strategies promoting walkability should incorporate equity-focused, age-friendly approaches to ensure benefits reach socioeconomically disadvantaged populations.

**Supplementary Information:**

The online version contains supplementary material available at 10.1007/s11524-026-01102-1.

## Introduction

Obesity remains a major global public health challenge, especially among older adults [1]. Defined by excess adiposity, obesity increases the risk of metabolic disorders, type 2 diabetes, cardiovascular disease, and premature death [2]. Its prevalence continues to rise globally [3]. In Europe, Spain ranks among the countries with the highest prevalence of obesity in this age group [4], with notable regional disparities. Southern regions, including Andalusia, report substantially higher rates compared to the East, North-East, and Central regions [5]. Together, the rising prevalences, uneven disease burden, and marked regional disparities are placing a growing strain on health-care systems [6].

Obesity disproportionately affects individuals from lower area-level socioeconomic status (SES), reflecting persistent structural inequalities across populations [7]. In Spain, these patterns are also evident: obesity shows the greatest relative inequality among major cardiovascular risk factors [8].

Walkability is the extent to which the built environment (e.g., population density, street connectivity, access to a mix of daily living amenities) supports walking [9]. Neighborhood walkability is a key environmental factor linked to healthier weight outcomes [10], although evidence among older adults remains limited and inconsistent [11–14].

Cities vary in SES and walkability from one neighborhood to another, potentially explaining why obesity rates vary from one area to another.

A recent systematic review found only tentative evidence that neighborhood walkability moderates socioeconomic inequalities in obesity [15]. Based on just two studies that examined this interaction, the findings were inconsistent: one reported no evidence of moderation [16], whereas the other reported a marginal attenuation of inequalities in highly walkable areas [17]. Critically, both studies relied on self-reported weight and did not include older adults. Consequently, we still lack clear evidence on whether walkability moderate area-level disparities in obesity among older adults, a question with important public-health implications. This study aimed to address this research gap by examining whether neighborhood walkability moderates the association between area-level SES and objectively measured adiposity indicators in older adults in Andalusian cities.

## Methods

### Study Population

We carried out a baseline cross-sectional analysis using data from a subsample of participants from a multicenter, parallel-group, randomized trial, carried out in Spain. The study included men and women aged 55 to 75 years with overweight or obesity (body mass index [BMI] 27–40 kg/m^2^) who met at least three metabolic syndrome criteria, including abdominal obesity, high blood pressure, high fasting glucose and triglycerides, and low high-density lipoprotein (HDL) cholesterol levels [18]. Details of the study design and protocol have been extensively described [19]. The study adhered to the Declaration of Helsinki for Medical Research involving human subjects and received approval from the institutional review boards of all recruiting centers. All participants provided written informed consent prior to enrollment. The study protocol was registered in the ISRCTN Registry (ISRCTN89898870).

For the present analysis, we included participants recruited from Andalusia (southern Spain) who reported a residential address within urban-area boundaries. We enrolled individuals between April 2014 and December 2016 from 32 primary care health centers near the research facilities associated with the original randomized trial previously conducted in Málaga, Córdoba, Granada, Sevilla and Jaén. The analysis used a baseline database compiled in December 2022 with 1,286 participants from the urban areas from these cities in Andalusia.

As this analysis was conducted using secondary data, post hoc power analyses were performed using the observed effect sizes for all presented associations between area-level socioeconomic status and adiposity-related variables included, in the available sample of 1286 participants. The power exceeded 80% for all analyzed associations.

Baseline residential addresses were geocoded to the street level with tidygeocoder (v1.0.5) using Google Maps and Mapbox [20]. We report our analyses in accordance with the Spatial Lifecourse Epidemiology Reporting Standards (ISLE-ReSt) guidelines (Supplementary Table S1) [21].

### Outcome Assessment

We assessed adiposity using BMI as a general adiposity measure, waist circumference (WC) and waist-to-hip ratio (WHR) as central measures, and a body shape index (ABSI) which quantifies central adiposity independently of body weight and height. Higher ABSI values indicate greater abdominal fat accumulation for a given BMI, capturing variability in fat distribution that BMI does not detect, a relevant property in populations with overweight or obesity, where BMI has limited discriminative capacity.

Trained personnel measured participants’ body weight (kg), height (cm), hip and waist circumferences (cm) in light clothing, and took two measurements. The repeated measurements were averaged. We computed BMI as weight (kg) divided by height squared (m^2^). We calculated WHR by dividing waist circumference by hip circumference, with both measurements in centimeters. We derived ABSI using the formula: waist circumference × weight^(−2/3) × height^(5/6), then multiplied the results by 1000 to simplify interpretation [22, 23].

### Exposure Assessment

**Fig. 1:**
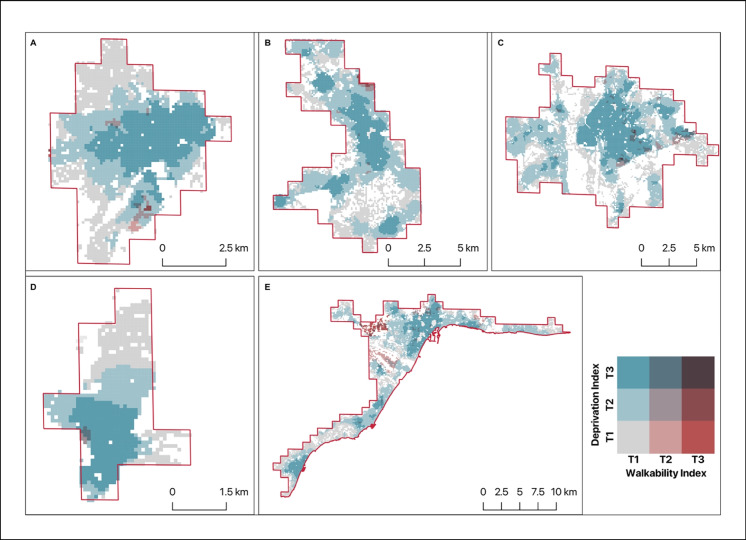
Citywide area-level SES and neighborhood walkability for Andalusian study recruited centers, Spain. Note: Panels show **A** Córdoba, **B** Granada, **C** Sevilla, **D** Jaén and **E** Málaga. A 100m‐resolution grid is shaded by tertiles of the Spanish Deprivation Index (T1 = least deprived, T2 = intermediate, T3 = most deprived; darker blues indicate higher deprivation), and by tertiles of walkability (T1 = least walkable, T2 = intermediate, T3 = most walkable; darker reds indicate higher walkability). The bivariate legend shows the joint distribution of deprivation and walkability tertiles. Census tract-level 2011 Spanish Deprivation Index ranged from −2.28 (least deprived) to 4.75 (most deprived). City-specific means (SD) were: −0.58 (1.03) for Córdoba, −0.48 (0.78) for Granada, −0.46 (1.04) for Sevilla, −0.36 (0.81) for Jaén, and −0.21 (1.07) for Málaga. Grid-level 2014 walkability ranged from −7.69 (least walkable) to 5.87 (most walkable). City-specific means (SD) were: −0.73 (2.72) for Córdoba, −0.86 (2.27) for Granada, −0.47 (2.56) for Sevilla, 0.02 (2.58) for Jaén, and −0.77 (2.39) for Málaga. The red line delimits the urban boundary used for the analysis. All maps share a common cartographic scale; individual scalebars are provided for reference

We identified area-level SES using deprivation measure from the 2011 Spanish Deprivation Index (IP2011), where higher IP2011 values indicate greater socioeconomic deprivation. The IP2011 is based on 2011 national census data to assess deprivation based on six area-level disadvantage indicators: proportion of manual and temporary workers, unemployment rate, insufficient overall education and among young adults (aged 16–29 years), and dwellings without internet access [24]. The IP2011 is the latest version and is freely available online [25] (Fig. 1).

### Potential Moderators: Neighborhood Walkability

We identified area-level neighborhood walkability using the *Global Healthy and Sustainable City Indicators*, an open science toolkit [26, 27] developed for a proof-of-concept analysis of spatial indicators for 25 cities across 19 countries and 6 continents [28]. The *Global Healthy and Sustainable City Indicators* software is freely available online [29].

We defined the urban study regions (Málaga, Córdoba, Granada, Sevilla and Jaén) using the *Global Human Settlements 2019 urban centres* layer [30] and calculated measures of local walkability for 100-m grids associated with 2020 population estimates [31]. Street networks and built environment features were extracted from OpenStreetMap using temporal data corresponding to the recruitment period (2014, 2015, 2016) [32] [32], Public transport schedule data were obtained from transport agencies in general transit feed specification (GTFS) format for the recruitment period.

Following the *Global Healthy and Sustainable City Indicators* software configuration for the “1000 City Challenge,” we calculated a local walkability index for each 100-m grid (Fig. 1). This index summed, for each city, equal-weighted standardized scores of three components: population density, street intersection density, and daily living score [33]. The daily living score was defined as the sum of equal-weighted binary access scores to a healthy food market, a convenience store, and a public transport stop within 500 m. In subsequent analyses, we examined both the composite local walkability index and its individual components (population density, street intersection density, and daily living score) as separate walkability measures to identify which specific built-environment features drove any observed effect modification.

### Covariate Information

We selected the covariates for the models by constructing a Directed Acyclic Graph (DAG), presented in the Supplementary Fig. 1 of the Supplement. Covariates were identified a priori as potential confounders based on a careful review of the relevant literature on the relationship between area-level SES, walkability, and adiposity [7]. In our conceptual model, walkability was treated as an effect modifier rather than a mediator of the SES-adiposity association. Empirical support for this specification is provided in the sensitivity analysis (Supplementary Table S5) and discussed further below.

We collected baseline sociodemographic and lifestyle covariates via a self-reported questionnaire. These included age, sex, highest achieved education, civil status, smoking, alcohol intake, physical activity, dietary adherence, and health status. We classified education as university, high school or equivalent, and primary school or less. Civil status was categorized as married, single or divorced, and widow/widower. Smoking status was grouped into never, former, or current smoker. We used a validated semi-quantitative food-frequency questionnaire to estimate alcohol intake (grams per day, g/d) [34]. Participants self-reported dietary adherence to an energy-restricted Mediterranean diet using the validated 17-item er-MEDAS questionnaire (score range: 0–17), with higher scores indicating greater adherence [35]. Leisure-time physical activity was assessed with the validated REGICOR questionnaire, estimating total energy expenditure in MET·min/week [36]. We assessed health status using the validated Spanish version of the SF-36 [37] question: “How would you rate your general health?” Participants reporting excellent, very good, or good health were grouped together, while those reporting fair or poor health comprised the second category.

### Statistical Analysis

We summarized participant characteristics by area-level SES, categorizing neighborhoods as lower or higher SES based on a median split of IP2011 census tract scores. Continuous variables were reported as median (p25; p75), while categorical variables were reported as counts and percentages.

Two-level linear regression models were fitted to examine the association between the area-level socioeconomic deprivation index (IP2011) and adiposity measures, while accounting for clustering of participants (level 1) within census areas (level 2) using the *lme4* package [38]. All models were adjusted for individual-level covariates selected a priori based on the DAG (Supplementary Fig. 1). Demographic confounders included age, sex, education, marital status, smoking status. Lifestyle covariates included alcohol consumption and self-reported health status, dietary adherence (er-MEDAS), physical activity (REGICOR MET·min/week). According to our DAG, dietary adherence and physical activity may partly lie on the causal pathway from area-level SES to adiposity. However, we retained them in the fully adjusted models for two reasons. First, both variables are also influenced by multiple confounders shared with the outcome (age, sex, education, health status), and their inclusion helps control residual confounding through these pathways. Second, adjusting for potential mediators attenuates the estimated SES-adiposity association toward the null; the persistence of significant effect modification under this stricter adjustment supports the robustness of the interaction findings. We acknowledge that this approach may underestimate the total effect of area-level SES on adiposity. To address potential multicollinearity, we calculated the Variance Inflation Factor (VIF) for each confounder, applying a strict threshold of 3; all VIF values were below this threshold [39].

To examine whether walkability modified the association between area-level SES and adiposity, we added an interaction term (IP2011 × walkability) to each mixed-effects model and assessed its overall significance with a Wald test (car::Anova) [40]. A significant Wald test suggests that the association between SES on adiposity measure differs across levels of walkability.

Finally, we conducted stratified analyses by tertiles of the walkability indicators (low, medium, high) to evaluate the consistency of SES associations across different walkability environments.

As a sensitivity analysis addressing the potential dual role of walkability as both mediator and effect modifier, we conducted a change-in-estimate analysis. We compared the IP2011 coefficient in models with and without walkability adjustment to assess whether the indirect pathway SES-Walkability-Adiposity attenuated the main exposure effect (Supplementary Table S5).

All statistical analyses were conducted using R software, version 4.2.3.

## Results

Participant characteristics and walkability measures by area-level SES are presented in Table 1. The overall sample included 1286 participants, classified into higher (*n* = 645) and lower (*n* = 641) SES categories based on their census tract IP2011 scores, ensuring an equal number of census tracts (but not participants) per SES category.

**Table 1:** Participant characteristics by area-level socioeconomic status

	**Area-level socioeconomic status**			
	**Overall (*n* = 1286)**	**Higher SES (*n* = 645)**	**Lower SES (*n* = 641)**	***p* value**
**Sociodemographic data**				
**Age years**^**#**^	65.0 (61.0; 68.0)	65.0 (61.0; 68.0)	65.0 (61.0; 68.0)	0.121
**Sex, *n* (%)**				0.434
Female	653 (51%)	320 (50%)	333 (52%)	
Male	633 (49%)	325 (50%)	308 (48%)	
**Education level, *n* (%)**				< 0.001
Primary school or less	682 (53%)	293 (45%)	389 (61%)	
High school or equivalent	349 (27%)	170 (26%)	179 (28%)	
University	255 (20%)	182 (28%)	73 (11%)	
**Civil status, *n* (%)**				0.360
Married	984 (77%)	504 (78%)	480 (75%)	
Single or divorced	157 (12%)	73 (11%)	84 (13%)	
Widow/Widower	144 (11%)	67 (10%)	77 (12%)	
**Self-reported health status, *n* (%)**				0.101
Excellent/very good/good	782 (61%)	407 (64%)	375 (59%)	
Fair/poor	494 (39%)	233 (36%)	261 (41%)	
**Lifestyle habits**				
**Smoking habit, *n* (%)**				0.022
Current smoker	187 (15%)	100 (16%)	87 (14%)	
Former smoker	505 (39%)	272 (42%)	233 (37%)	
Never smoker	587 (46%)	270 (42%)	317 (50%)	
**Alcohol consumption, g/d**^**#**^	4 (1; 14)	6 (1; 16)	4 (1; 12)	0.003
**17-item MedDiet ­score**^**#**^	9.00 (7.00; 11.00)	9.00 (7.00; 11.00)	9.00 (7.00; 11.00)	0.288
**Total physical activity, MET min/week**^**#**^	1675 (671; 3,245)	1538 (615; 3,077)	1734 (722; 3357)	0.108
**Anthropometric data**				
**BMI, (kg/m**^**2**^**)**^**#**^	32.3 (29.8; 35.3)	32.3 (29.8; 35.0)	32.4 (29.9; 35.7)	0.317
**Obesity (BMI ≥ 30)**	952 (74%)	475 (74%)	477 (74%)	0.801
**Waist circumference (cm)**^**#**^	107 (100; 114)	106 (100; 114)	107 (101; 115)	0.377
**Waist-to-hip ratio**^**#**^	0.98 (0.92; 1.03)	0.97 (0.92; 1.03)	0.98 (0.92; 1.03)	0.798
**ABSI**^**#**^	82.6 (79.6; 85.7)	82.5 (79.3; 85.5)	82.6 (79.9; 85.8)	0.326
**Environment**				
**IP2011**	−0.18 (−0.85; 0.53)	−0.85 (−1.22; −0.45)	0.56 (0.05; 1.04)	< 0.001
**Population density**	11,782 (6,506; 15,485)	12,731 (7,260; 16,493)	10,909 (6,411; 14,465)	< 0.001
**Street intersection density**	197 (175; 219)	189 (171; 219)	201 (178; 218)	< 0.001
**Daily living score**	2.00 (1.47; 3.00)	2.00 (1.50; 3.00)	2.00 (1.43; 3.00)	0.931
**Local walkability index**	2.39 (0.92; 3.57)	2.40 (0.71; 3.81)	2.36 (1.17; 3.39)	0.528
**Local walkability index tertile**				< 0.001
**Low**	429 (33%)	236 (37%)	193 (30%)	
**Medium**	429 (33%)	182 (28%)	247 (39%)	
**High**	428 (33%)	227 (35%)	201 (31%)	

Note: data are *n* (%) for categorical variables. ^**#**^Unless specified, quantitative data are presented as median (p25; p75). Lower and higher area-level socioeconomic status (SES) categories were created using a median split of census tract IP2011 scores.

A body shape index (ABSI) was calculated as waist circumference × weight^−2/3^ × height^5/6^. ABSI was multiplied by 1000 to facilitate interpretation

^1^Notes on scales: the MedDiet scores range between 0 and 17. Higher MedDiet scores represent higher adherence to the Mediterranean diet

Abbreviations: *SD* standard deviation; *MedDiet* Mediterranean diet; *BMI* body mass index; *ABSI* a body shape index; *IP2011* deprivation index 2011; Daily Living Score ranges from 0 to 3, summing equal-weighted binary access scores to a healthy food market, a convenience store, and a public transport stop within 500 m.

By study design, all participants had overweight or obesity. Anthropometric measures were consistent across SES categories. Obesity prevalence (BMI ≥ 30 kg/m^2^) was 74.0% overall, 73.6% in higher-SES areas and 74.4% in lower-SES areas, indicating no SES differences in obesity. Among all participants, the median body-mass index was 32.3 kg/m^2^ (IQR 29.8–35.3), with nearly identical medians across SES categories: 32.3 kg/m^2^ (IQR 29.8–35.0) in higher-SES areas, and 32.4 kg/m^2^ (IQR 29.9–35.7) in lower-SES areas. Median waist circumference differed only marginally by neighborhood SES: 106 cm (IQR 100–114) in higher-SES areas versus 107 cm (101–115) in lower-SES areas. The waist-to-hip ratio showed virtually identical medians (0.97 vs 0.98) with identical IQRs (0.92–1.03). The ABSI body-shape index overlapped completely between groups, with a median of 82.6 in both higher-SES [82.5 (79.3–85.5)] and lower-SES [82.6 (79.9–85.8)] areas.

Median age was 65 years in both groups. The proportion of females was slightly higher in the lower-SES areas (52%) compared to higher SES areas (50%). Educational attainment was lower in the lower-SES areas, with 61% having primary education or less, compared with 45% in higher-SES areas. Marital status distribution was comparable between groups.

Self-reported fair or poor health was more common in lower-SES areas (41%) than higher SES areas (36%). Smoking habits differed modestly, with a greater proportion of never smokers in lower SES (50%) versus higher SES (42%). Median alcohol consumption was lower in the lower-SES group (4 g/d) than in the higher SES group (6 g/d). Mediterranean diet adherence scores and physical activity levels were comparable across SES groups.

By design, the lower-SES group had higher IP2011 deprivation scores. Walkability index and its components, including street intersection density and daily living scores, showed minor differences between SES groups.

Supplementary Table S2 shows Pearson correlation coefficients between studied variables. IP2011 showed weak but significant negative correlations with population density (*r* = −0.07, *p* < 0.01) and positive correlations with street intersection density (*r* = 0.11, *p* < 0.001). The local walkability index showed strong positive correlations with its three components population density (*r* = 0.78), street intersection density (*r* = 0.69), and daily living score (*r* = 0.68), all *p* < 0.001; and a weak positive correlation with waist circumference (*r* = 0.09, *p* < 0.01).

Supplementary Table S2 summarizes descriptive statistics of each walkability measure across tertiles of the local walkability index. All component indicators increased median values across walkability tertiles. Local walkability index rises from 0.41 in low-walkable areas to 4.34 in high-walkable areas, population density rises from −0.10 to 1.98, street intersection density rises from −0.31 to 1.48, and daily living score rises from −0.24 to 1.81.

Table 2 presents the significance of interactions between area-level SES and walkability index and its components on adiposity outcomes, assessed via Wald tests. Local walkability index showed significant interactions with SES for waist circumference (*χ*^2^ = 4.60, p = 0.0320), waist-to-hip ratio (*χ*^2^ = 4.51, *p* = 0.0337), and ABSI (*χ*^2^ = 4.01, *p* = 0.0451). Significant interactions also were observed between SES and population density for waist circumference (*χ*^2^ = 10.46, *p* = 0.0012) and ABSI (*χ*^2^ = 6.76, *p* = 0.0093). Street intersection density was significantly interactive with SES only for waist-to-hip ratio (*χ*^2^ = 5.10, *p* = 0.0239). In contrast, no significant interactions were found for daily living score across adiposity outcomes, and no significant interactions were found between area-level SES and any walkability measures on BMI.

**Table 2:** Significance of the interaction between area-level SES and walkability measures: results of Wald tests

	**Wald chi-square**	***p* value**
**BMI**		
Local walkability index	0.17	0.6795
Population density	2.54	0.1112
Street intersection density	0.03	0.8650
Daily living score	0.45	0.5017
**Waist circumference**		
Local walkability index	4.60	**0.0320**
Population density	10.46	**0.0012**
Street intersection density	0.76	0.3837
Daily living score	0.02	0.8943
**Waist-to-hip ratio**		
Local walkability index	4.51	**0.0337**
Population density	2.00	0.1577
Street intersection density	5.10	**0.0239**
Daily living score	0.02	0.8856
**ABSI**		
Local walkability index	4.01	**0.0451**
Population density	6.76	**0.0093**
Street intersection density	0.24	0.6266
Daily living score	0.09	0.7682

Note: bold figures denote significant interaction. Models are adjusted for age, sex, education, civil status, smoking, alcohol intake, physical activity, dietary adherence, and health status and corrected for census areas level clustering

Abbreviations: *BMI* body mass index; *ABSI* a body shape index

Table 3 presents the associations between area-level SES and adiposity measures, stratified by tertiles of walkability and its components. Regression coefficients (B) represent the average change in each adiposity measure per one-SD increase in area-level deprivation (IP2011). For BMI, no significant associations with SES were found across walkability tertiles. Stratified analyses revealed that population density was the walkability component showing the strongest evidence of effect modification. In areas with higher population density, IP2011 was significantly associated with larger waist circumference (B = 1.25, 95% CI 0.23 to 2.27) and higher ABSI (B = 0.86, 95% CI 0.30 to 1.41). The local walkability index and street intersection density showed associations in the same direction in the highest tertile, but confidence intervals included the null (waist circumference: B = 0.96, 95% CI −0.18 to 2.09 for the walkability index; B = 1.14, 95% CI −0.12 to 2.41 for street intersection density). Waist-to-hip ratio showed no consistent pattern of association with area-level SES across walkability strata, with coefficients close to zero and confidence intervals including the null in all tertiles. No consistent pattern of effect modification was observed for the daily living score.

**Table 3:** Associations of area-level SES with adiposity stratified by tertiles of the walkability measures

Walkability measure	B (95% CI)		
	**Lower**	**Medium**	**Higher**
**BMI**			
Local walkability index	0.11 (−0.26, 0.49)	−0.28 (−0.65, 0.10)	0.09 (−0.25, 0.43)
Population density	−0.27 (−0.73, 0.18)	−0.01 (−0.38, 0.35)	0.02 (−0.30, 0.33)
Street intersection density	0.02 (−0.35, 0.39)	−0.33 (−0.72, 0.06)	0.09 (−0.29, 0.46)
Daily living score	0.15 (−0.20, 0.50)	−0.12 (−0.52, 0.28)	−0.24 (−0.59, 0.11)
**Waist circumference**			
Local walkability index	0.16 (−0.89, 1.21)	−0.43 (−1.42, 0.55)	0.96 (−0.18, 2.09)
Population density	−0.73 (−2.01, 0.56)	−0.30 (−1.22, 0.63)	1.25 (0.23, 2.27)*
Street intersection density	0.16 (−0.83, 1.15)	−0.82 (−1.86, 0.21)	1.14 (−0.12, 2.41)
Daily living score	0.54 (−0.43, 1.50)	0.19 (−0.95, 1.33)	−0.32 (−1.39, 0.75)
**Waist-to-hip ratio**			
Local walkability index	−0.00 (−0.01, 0.00)	0.00 (−0.00, 0.01)	0.01 (−0.00, 0.01)
Population density	0.00 (−0.01, 0.01)	−0.00 (−0.01, 0.00)	0.01 (−0.00, 0.01)
Street intersection density	−0.00 (−0.01, 0.00)	0.00 (−0.00, 0.01)	0.00 (−0.00, 0.01)
Daily living score	−0.00 (−0.01, 0.01)	0.00 (−0.00, 0.01)	0.00 (−0.01, 0.01)
**ABSI**			
Local walkability index	0.15 (−0.31, 0.60)	0.16 (−0.32, 0.65)	0.44 (−0.13, 1.02)
Population density	0.19 (−0.23, 0.61)	−0.09 (−0.50, 0.33)	0.86 (0.30, 1.41)**
Street intersection density	0.14 (−0.30, 0.59)	0.01 (−0.49, 0.51)	0.52 (−0.08, 1.12)
Daily living score	0.17 (−0.28, 0.63)	0.46 (−0.07, 0.98)	0.14 (−0.38, 0.65)

Note: *p < 0.05, **p < 0.01, ***p < 0.001, bold figures denote significant coefficients

Models are adjusted for age, sex, education, civil status, smoking, alcohol intake, physical activity, dietary adherence, and health status and corrected for census areas level clustering. Regression coefficients correspond to one SD increment in IP2011 score

Abbreviations: *BMI* body mass index; *ABSI* a body shape index; *CI* confidence interval

Supplementary Table S4 further explores these findings by comparing deprivation strata levels across strata levels of neighborhood walkability. It shows how lower, medium, and higher local walkability and its components differ in IP2011 scores within lower and higher SES groups. For the local walkability index, the SES contrast in deprivation was consistent across tertiles: the lower-SES group showed higher deprivation (mean IP2011 ≈ 0.6 to 0.7, SD ~ 0.7) and the higher-SES group showed lower deprivation (mean IP2011 ≈ −0.9, SD ~ 0.4 to 0.5). Overall means ranged from −0.2 to −0.2 with SD ~ 1.0. Similar stability in SES contrasts was observed for the individual walkability components (population density, street intersection density, and daily living score).

## Discussion

In this study of 1,286 Mediterranean older adults with overweight/obesity, we found no overall differences in adiposity across area-level SES. These findings contrast with prior research reporting showing higher adiposity in more deprived areas [7]. Nevertheless, the absence of an overall SES difference may be partly explained by the study’s inclusion criteria, which selected participants with overweight/obesity and metabolic syndrome [19], resulting in a relatively homogeneous sample with elevated adiposity regardless of neighborhood SES. However, neighborhood walkability context mattered; walkability modified this association. Wald tests for the SES × walkability interaction terms were statistically significant for waist circumference and ABSI (Table 2), indicating that the relationship between area-level deprivation and central adiposity differed across levels of neighborhood walkability. Stratified analyses (Table 3) revealed that in neighborhoods with higher population density, residents of more deprived areas had larger waist circumference and higher ABSI values compared with those in less deprived areas. In areas with moderate or low walkability, these SES differences were attenuated and not statistically significant. Population density thus emerged as the walkability component most strongly shape socioeconomic disparities in abdominal adiposity among older adults with overweight/obesity.

Our findings contribute to an emerging but inconsistent body of literature. Prior research has consistently shown higher obesity prevalence in socioeconomically disadvantaged areas, reflecting deep-rooted structural inequalities [7]. Whether neighborhood walkability modifies these inequalities remains unclear [15]. Among the few studies examining the interaction between SES and walkability, one study found no significant moderation effect [16], while McCormack et al. reported that SES-related disparities in waist circumference were more pronounced in less walkable neighborhoods and diminished in more walkable ones. However, this interaction was only marginally significant for waist circumference and non-significant for BMI and waist-to-hip ratio [17]. By contrast, our findings diverge from those of McCormack et al., as we observed stronger SES gradients in central adiposity within neighborhoods characterized by high walkability and high population density. Differences in age, objectively measured outcomes, and a Mediterranean urban form may explain the divergence. Unlike most prior research, we focus on older adults, a group underrepresented in urban health research, with objective adiposity measures and living in Mediterranean Spanish cities, where urban form, social dynamics, and lifestyle differ from those previously studied populations.

Our findings suggest that in Mediterranean cities with a high population density, the walkability component showing the strongest evidence of effect modification, can amplify deprivation-related differences in central adiposity, whereas BMI remains insensitive. Although Wald tests indicated significant SES × walkability interactions for the composite index, stratified analyses showed that the strongest and only statistically significant evidence of effect modification was observed for population density. The local walkability index and street intersection density showed consistent but non-significant trends, suggesting that population density may be the primary driver of walkability-related moderation in this context. This pattern indicates that current urban forms do not deliver uniform benefits for older adults in deprived areas of these Mediterranean cities. Dense areas give people more chances to be active. However, in deprived neighborhoods, barriers make activity harder, and other exposures (heat, noise, air pollution, unhealthy food) reduce the benefits and widen adiposity inequalities [10, 41].

Our findings hold key implications for ageing cities. The lack of a strong overall deprivation gradient supports population-wide obesity prevention in older adults with overweight/obesity. At the same time, walkability can modify obesity inequalities; in our Mediterranean context it intensified SES gaps in central adiposity, underscoring the need for health-promoting, age-friendly walkability in deprived neighborhoods.

Urban planners should recognize that walkability benefits older adults but conventional, density-driven forms do not automatically deliver equity. Investments must prioritize deprived neighborhoods and deliver health-promoting, age-friendly walkability. Integrating health considerations into urban design “health in all policies” is essential, with a life-course perspective emphasizing walkability from early to late life.

This study’s strengths include objective measurement of adiposity and detailed geospatial assessment of neighborhood walkability across multiple cities, reducing common bias in self-reported data. Focusing on older adults with overweight/obesity addresses an understudied high-risk group. The use of multilevel modeling accounts for clustering by neighborhood, and adjustment for relevant confounders identified a priori via a directed acyclic graph strengthens internal validity. Multiple walkability components allowed comprehensive assessment of the built environment. A sensitivity analysis confirmed that walkability did not meaningfully mediate the SES-adiposity association, supporting its role as an effect modifier rather than a pathway variable.

Limitations include its cross-sectional design, precluding causal inference and subject to residential self-selection bias. The specific study population, older adults already overweight or obese with metabolic syndrome, may limit generalizability and may compress observed deprivation gradients. Results from urban Andalusian settings may not extend to rural areas or other cultural contexts. Exposure assessment often assumes that the neighborhood where a person lives accurately represents their environmental exposure. However, this does not take into account their daily movements and travel patterns, which can lead to inaccuracies—an issue known as the uncertain geographic context problem. Quality aspects of walkability (e.g., sidewalk condition, safety) were not measured. Potential residual confounding by unmeasured factors remains. Marginal significance of some interactions suggests caution, though consistent patterns support robustness. Longitudinal studies are needed to confirm findings.

Among older Mediterranean adults with overweight/obesity, neighborhood walkability modified area-level socioeconomic inequalities in central adiposity. While obesity prevalence is high regardless of neighborhood deprivation, in high-walkability settings, greater deprivation was linked to larger waist circumference and higher ABSI, whereas gradients were weak or absent in less walkable areas. These results suggest that urban environments may intensify deprivation-related differences in abdominal adiposity. Urban policy should prioritize health-promoting, age-friendly walkability, particularly in deprived neighborhoods.

Future longitudinal research should clarify pathways and guide evidence-based urban health policies aimed at fostering equitable healthy aging.

## Supplementary Information

Below is the link to the electronic supplementary material.

ESM 1 (DOCX 1.28 MB)

## Data Availability

The study protocol of PREDIMED Plus, including its statistical analysis plan for the main study, has been published earlier [42]. The protocol can also be downloaded from https://www.predimedplus.com/. The datasets generated and analyzed during the current study are not publicly available due to data regulations and ethical reasons. However, collaboration for data analyses can be requested by sending a letter to the PREDIMED-Plus Steering Committee (predimed_plus_scommittee@googlegroups.com). The request will then be passed to all the members of the PREDIMED-Plus Steering Committee for deliberation.
