# Supplementary material for: Area-Level Socioeconomic Inequalities in Adiposity among Older Adults: the Moderating Effect of Neighborhood Walkability
Source: J Urban Health. 2026 Jun 12;103(3):600–12. doi: 10.1007/s11524-026-01102-1 (PMC13315632; doi:10.1007/s11524-026-01102-1)
Supplement: Supplementary file 1 — (DOCX 1.28 MB) [file 11524_2026_1102_MOESM1_ESM.docx]

**Supplementary Information:**

**Full title:** Area-level socioeconomic inequalities in adiposity among older adults: the moderating effect of neighborhood walkability.

Antoni Colom, Napoleón Pérez-Farinós, Francisco Javier Barón-López, Maurici Ruiz-Pérez, Marc Dominguez Mallafré, Xavier Delclòs-Alió, Antonio Garcia-Rios, Aurora Bueno-Cavanillas, Francisco J. Tinahones, Víctor Urbano-Fernández, José J. Gaforio, Jordi Salas-Salvadó, Montserrat Fitó, Julia Wärnberg.

Table of content

[Supplementary Table S1. ISLE-ReSt checklist. 1](#_Toc211106680)

[Supplementary Table S2. Correlation coefficients between variables. 4](#_Toc211106681)

[Supplementary Table S3. Median (min, max) of the walkability measures for each walkability measure tertile. 5](#_Toc211106682)

[Supplementary Table S4. Mean (SD) of IP2011 in lower and higher SES areas for each walkability tertile ants its components 5](#_Toc211106683)

[Supplementary Figure 1. Directed Acyclic Graph regarding the expected associations between area-level SES and adiposity. 6](#_Toc211106684)

# **Supplementary Table S1.** ISLE-ReSt checklist.

|  | Item No | Recommendation | Page No |
| --- | --- | --- | --- |
| Title and abstract | | | |
| Title | 1 | Indicate the primary exposure variable(s) and main outcome variable(s) | 1 |
| Abstract | 2 | Provide in the abstract an informative and balanced summary of objectives, methods (including study design, primary exposure variable(s) of interest, including data sources, and main outcome variable(s) of interest), results (association between primary exposures and main outcomes of interest), and conclusions | 3 |
|  |  |  |  |
| Introduction | | | |
| Background/  Rationale | 3 | Explain the scientific background and rationale for the investigation being reported; provide a specific conceptual or theoretical framework/description of links between environmental and health variables included | 6-7 |
| Objectives | 4 | State specific objectives, including pre-specified hypotheses | 7 |
| Methods | | | |
| Study design | 5 | Present full description of study design, including a clear rationale for the spatial scale at which exposure was measured | 7-13 |
| Setting | 6 | Describe the setting, locations, and relevant dates (e.g., periods of recruitment, exposure, follow-up, and data collection) | 7-13 |
| Participants/  Sample size | 7 | Give the eligibility criteria, and the sources and methods of selection of participants; describe methods of follow-up and how the study size was determined; describe approaches to link participant data to spatial locations (e.g., method, reference data set, coordinate systems, and software package used to geocode, % of participants geocoded to an address and/or a predefined areal unit) | 7-13 |
| Variables | 8 | Clearly define all outcomes, exposures, predictors, potential confounders, and effect modifiers; give diagnostic criteria, if applicable | 7-14 |
| Health data sources/  measurement | 9 | For each variable of interest, give sources of data and details of methods of assessment (measurement); describe comparability of assessment methods if there is more than one group | 7-14 |
| Bias | 10 | Describe any efforts to address potential sources of bias | 7-15 |
| Quantitative variables | 11 | Explain how quantitative variables were handled in the analyses; describe which groupings were chosen and why, if applicable | 13-15 |
| Spatial data | 12 | GIS— Give data source (URL if open source data), time of collection, spatial resolution, and processing methods  RS— Give the name and spatiotemporal resolutions of satellite sensors from which images are derived, dates images were taken, and any preprocessing procedures; provide the processing method and/or the citation for RS products  GPS(+accelerometer)— Give the name, model, and measurement error of all devices, the interval, period, and duration of data collection  Smartphone app— Give the details (e.g., measurement error) of the device used, the name and platform of the app used, the frequency and recording of location updates, and the method, period, and duration of data collection (e.g., food image-taking)  Other sensor data— Specify the technology, developer, detailed usage and measurement error of the sensor, the frequency and recording of location updates, and the method, period, and duration of data collection | 9 |
| Spatial methods | 13 | GIS— Describe the method, justification, and software package used to produce spatial factors (e.g., buffer type and radius for defining contextual areas, codes used for extracting features from commercial data sets) and match spatial factors with health data  RS— Describe the method and software package used to (pre)process images/products, produce spatial factors, and match spatial factors with health data  GPS(and accelerometer) and Smartphone app— Give the numbers of valid days and hours per day required for a valid day, criteria for non-wear, and accelerometer count thresholds for intensity of activity, and the methods used to (pre)process tracked location data, define activity space, and validate the collected data (e.g., travel diary, dietary recall, and food frequency questionnaire) | 9-15 |
| Statistical methods | 14 | Describe all statistical methods (e.g., those used to control for confounding, clustering, endogeneity, and spatial autocorrelation), any methods used to examine subgroups and interactions, and any sensitivity analyses, including spatial inspection of residuals from models; explain how missing data, outliers, and loss to follow-up were addressed | 14-15 |
| Results | | | |
| Participants | 15 | Consider a flow diagram to report numbers of individuals at each stage of study and reasons for non-participation at each stage |  |
| Descriptive data | 16 | Give characteristics of study participants (e.g., sociodemographic, geographical, clinical) and information on exposures | Table 1 |
| Outcome data | 17 | Report numbers of outcome events or summary measures over time | 15-25 |
| Main results | 18 | Give unadjusted estimates and, if applicable, confounder-adjusted estimates and their precision (e.g., 95% confidence interval); make clear which confounders were adjusted for and why they were included; report category boundaries when continuous variables were categorized | 15-25 |
| Other analyses | 19 | Report other analyses done (e.g., subgroup, interaction, mediation, and sensitivity analyses) and spatial autocorrelation diagnostics | 15-25 |
| Discussion | | | |
| Key results | 20 | Summarize key results with reference to study objectives | 26 |
| Interpretation | 21 | Give a cautious overall interpretation of results considering objectives, limitations, multiplicity of analyses, results from similar studies, and other relevant evidence | 26-29 |
| Limitations | 22 | Describe limitations of the study (e.g., limitations of spatial data and methods used, temporal mismatches between health and spatial data, different spatial data sources at different time points, exposure misclassification issues, extent of reflecting real environmental exposure, potential direction and magnitude of bias, the uncertain geographic context problem, the neighborhood effect averaging problem, spatial and temporal non-stationarity, neighborhood self-selection, selective daily mobility bias, and selective migration) | 26-29 |
| Generalizability | 23 | Describe the generalizability (external validity) of the study results | 26-29 |
| Other information | | | |
| Source of funding | 24 | Give the source of funding and the role of the funders for the present study | 35 |
| Conflict of interest | 25 | Describe any potential for conflict of interest of study contributors in accordance with journal policy | 35 |
| Data sharing statement | 26 | Describe which data could be shared and how to access data (including codes of processing files). | 35 |
| GIS– Geographic Information Systems; RS– remote sensing; GPS– Global Positioning Systems. | | | |

# **Supplementary Table S2.** Correlation coefficients between variables.

|  | **BMI, (kg/m^2^)** | **Waist circumference (cm)** | **Waist-to-hip ratio** | **ABSI** | **Population density** | **Street intersection density** | **Daily living score** | **Local walkability index** |  |
| --- | --- | --- | --- | --- | --- | --- | --- | --- | --- |
| **IP2011** | 0.02 | 0.01 | -0.01 | 0.03 | -0.07** | 0.11*** | -0.02 | 0 |  |
| **BMI, (kg/m^2^)** | — | 0.69*** | 0.07* | -0.09*** | 0.06* | 0.06* | -0.04 | 0.03 |  |
| **Waist circumference (cm)** |  | — | 0.58*** | 0.6*** | 0.14*** | 0.08** | -0.03 | 0.09** |  |
| **Waist-to-hip ratio** |  |  | — | 0.65*** | -0.01 | 0.06* | -0.02 | 0.01 |  |
| **ABSI** |  |  |  | — | 0.17*** | 0.08** | 0.01 | 0.12*** |  |
| **Population density** |  |  |  |  | — | 0.34*** | 0.29*** | 0.78*** |  |
| **Street intersection density** |  |  |  |  |  | — | 0.17*** | 0.69*** |  |
| **Daily living score** |  |  |  |  |  |  | — | 0.68*** |  |
| **Local walkability index** |  |  |  |  |  |  |  | — |  |
| Note: * p < .05, ** p < .01, *** p < .001  Abbreviations: BMI = body mass index; ABSI = a body shape index; IP2011 = deprivation index 2011; | | | | | | | | | |

# **Supplementary Table S3**. Median (min, max) of the walkability measures for each walkability measure tertile.

| **Walkability measure** | **Lower**  **walkability** | **Medium**  **walkability** | **Higher**  **walkability** |
| --- | --- | --- | --- |
| **Local walkability index** | 0.41 (-4.19, 1.4) | 2.39 (1.43, 3.2) | 4.34 (3.2, 6.63) |
| **Population density** | -0.1 (-1.33, 0.25) | 0.9 (0.25, 1.46) | 1.98 (1.46, 2.79) |
| **Street intersection density** | -0.31 (-1.77, 0.21) | 0.67 (0.21, 1.1) | 1.48 (1.1, 2.56) |
| **Daily living score** | -0.24 (-1.48, 0.53) | 0.83 (0.53, 1.35) | 1.81 (1.36, 2.24) |

# **Supplementary Table S4**. Mean (SD) of IP2011 in lower and higher SES areas for each walkability tertile ants its components

|  | SES | Lower walkability | Medium walkability | Higher walkability |
| --- | --- | --- | --- | --- |
|  |  | IP2011 | IP2011 | IP2011 |
| Local walkability index | Low | 0.7 (0.7) | 0.7 (0.7) | 0.6 (0.7) |
|  | High | -0.9 (0.5) | -0.8 (0.5) | -0.9 (0.4) |
|  | Overall | -0.2 (1.0) | 0.0 (1.0) | -0.2 (1.0) |
| Population density | Low | 0.6 (0.6) | 0.6 (0.7) | 0.8 (0.8) |
|  | High | -0.8 (0.4) | -1.0 (0.5) | -0.9 (0.5) |
|  | Overall | 0.0 (0.9) | -0.2 (1.0) | -0.2 (1.1) |
| Street intersection density | Low | 0.7 (0.8) | 0.6 (0.6) | 0.6 (0.6) |
|  | High | -0.9 (0.5) | -0.9 (0.4) | -0.9 (0.4) |
|  | Overall | -0.2 (1.0) | -0.1 (0.9) | -0.0 (0.9) |
| Daily living score | Low | 0.8 (0.7) | 0.6 (0.6) | 0.6 (0.7) |
|  | High | -0.9 (0.5) | -0.9 (0.5) | -0.9 (0.5) |
|  | Overall | -0.1 (1.0) | -0.1 (0.9) | -0.2 (1.0) |

# **Supplementary Table S5**. Change in estimate analysis: IP2011 coefficient with and without walkability adjustment.

| **Outcome** | **Walkability_adj** | **Beta_without_walk** | **SE_without** | **Beta_with_walk** | **SE_with** | **Pct_change** |
| --- | --- | --- | --- | --- | --- | --- |
| BMI | Local walkability index | -0.0519 | 0.1082 | -0.0516 | 0.1081 | 0.57 |
| Waist | Local walkability index | 0.2104 | 0.3207 | 0.1828 | 0.3153 | 13.12 |
| WHR | Local walkability index | 0.001 | 0.0017 | 0.0011 | 0.0017 | -6.71 |
| ABSI | Local walkability index | 0.2614 | 0.1522 | 0.2446 | 0.1492 | 6.4 |

# **Supplementary Figure 1.** Directed Acyclic Graph regarding the expected associations between area-level SES and adiposity.

| 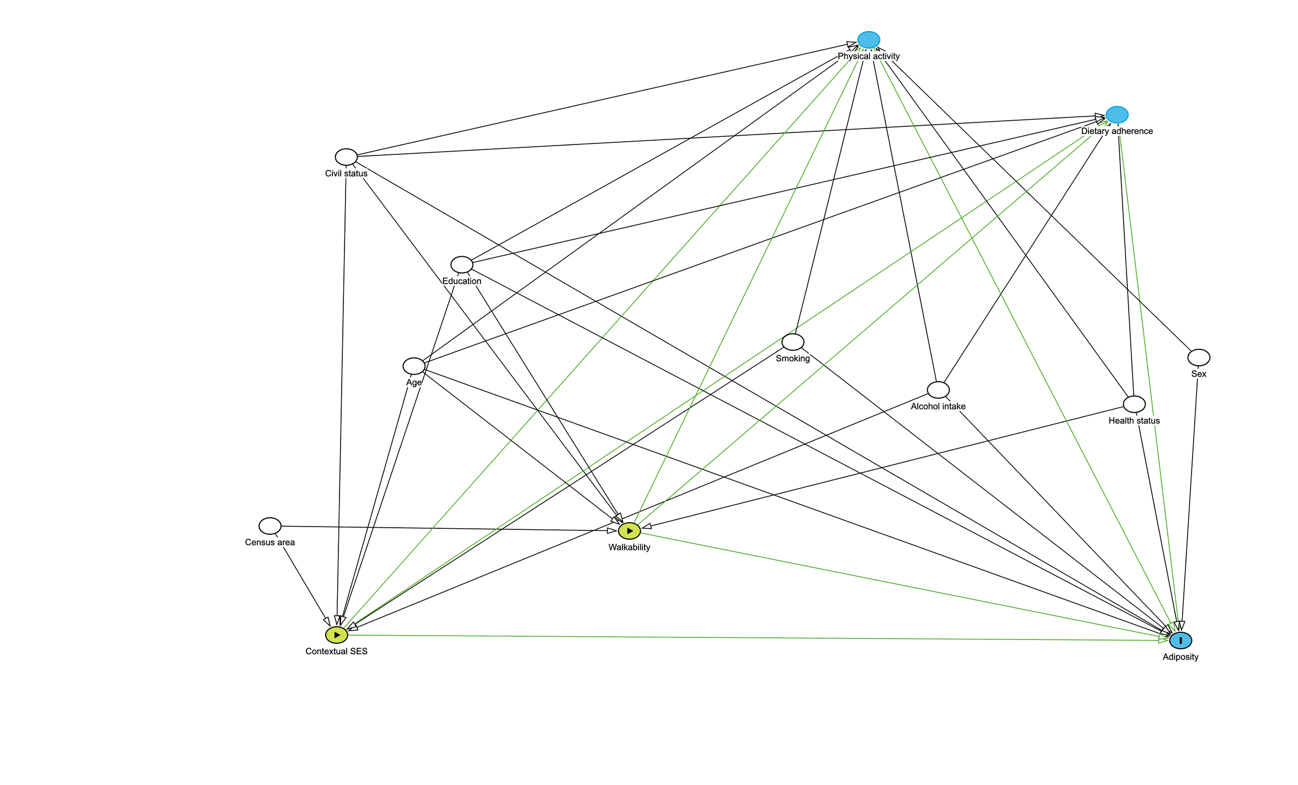 |
| --- |
| Note: Minimal sufficient adjustment sets containing Age, Alcohol intake, Census area, Civil status, Education, Health status, Sex, Smoking for estimating the total effect of contextual SES x Walkability on Adiposity. |
